# Information-Driven Rule Reduction in Belief Rule Bases for Complex System Modeling

**DOI:** 10.3390/e28050578

**Published:** 2026-05-21

**Authors:** Xingzhi Liu, Haolan Huang, Yingmei Li, Zida Xia, Shutong Zhao

**Affiliations:** 1 The School of Computer Science and Information Engineering, Harbin Normal University, Harbin 150025, China; 2 Faculty of Arts and Social Sciences, University of Sydney, City Road, Darlington, NSW 2008, Australia

**Keywords:** belief rule base, adaptive rule reduction, sensitivity analysis, industrial safety prediction

## Abstract

In the analysis of complex engineering systems, managing uncertainty and optimizing information processing structures are critical for reliable state prediction. The Belief Rule Base (BRB) provides a powerful machine learning approach for integrating expert knowledge with uncertain information. However, mitigating the combinatorial complexity of BRBs through conventional structure simplification often causes unintended information loss, introducing systematic prediction biases that undermine reliability. To address the trade-off between system complexity and modeling accuracy, this study proposes an adaptive belief rule base framework integrating sensitivity analysis with posterior consistency calibration (BRB-ARR). First, an information-driven rule screening mechanism is developed to dynamically determine the pruning threshold based on optimized Mean Square Error (MSE) fluctuations. This method effectively filters redundant rules while avoiding the cognitive biases associated with fixed empirical values. Second, a low-dimensional optimization process is employed to readjust the parameter vector, significantly enhancing computational efficiency. Finally, a posterior calibration module is introduced to compensate for the systematic biases caused by dimensionality reduction, strictly preserving the interpretability of the core inference architecture. To validate the effectiveness of the proposed framework, experimental evaluations are conducted on petroleum pipeline networks and liquid propellant launch vehicles. In the petroleum pipeline scenario, the rule base scale is reduced by over 60 percent from 56 to approximately 20 rules, while the parameter dimensionality decreases from 338 to 122. Compared to the conventional model, the mean squared error is reduced from 0.5291 to 0.3619. Furthermore, in the liquid propellant launch vehicle case, the model achieves a prediction accuracy of 98.57 percent with a mean squared error of 0.00029 while reducing the rule scale from 441 to 109. These results demonstrate that the BRB-ARR model effectively balances structural compactness with high precision prediction, offering a novel approach to uncertainty modeling in intelligent systems.

## 1. Introduction

Industrial safety prediction has emerged as a critical research focus in domains such as intelligent manufacturing, energy transportation, and process monitoring [1]. In practical high-risk industrial environments, predictive models are required not only to achieve satisfactory accuracy but also to deliver interpretable reasoning processes and reliable decision-making support. This requirement is especially critical in safety-critical infrastructure scenarios, where inaccurate or unreliable predictions may lead to economic losses, environmental pollution, and even operational accidents.

Existing data-driven prediction methods, such as deep neural networks (DNNs), support vector regression (SVR), and ensemble learning models, have demonstrated outstanding performance in modeling complex industrial systems owing to their powerful nonlinear mapping capabilities. Gholaminejad et al. proposed a deep neural network (DNN) method based on an autoencoder architecture, which effectively addressed the difficulty of fault feature extraction for induction motors under complex interference conditions and significantly improved diagnostic reliability [2]. Che et al. developed a hybrid prediction model that integrates the slime mold algorithm with support vector regression (SMA-SVR), thereby overcoming the difficulty of parameter optimization caused by small-sample data and high-dimensional features in predicting the mechanical properties of industrial low-alloy steels [3]. Al-Andoli et al. proposed a parallel ensemble learning framework that fuses base classifiers from multilayer deep networks, thereby addressing the poor generalization capability of a single data-driven model in condition monitoring of complex industrial machinery [4]. Madathil et al. noted that such data-driven methods are essentially “black-box” models whose internal decision-making mechanisms lack transparency and are therefore unable to provide intuitive causal explanations at the rule level, severely undermining their trustworthiness and reliability in high-risk industrial safety monitoring scenarios [5].

However, these methods are inherently black-box models, and their internal decision logic lacks transparency, making it difficult to provide intuitive explanations at the rule level. In the high-risk domain of industrial safety, this lack of interpretability not only weakens the credibility of prediction results but also prevents the effective integration of domain experts’ prior knowledge and uncertain information, thereby limiting their practical deployment in critical engineering scenarios.

In contrast, model-driven methods based on physical mechanisms possess explicit physical meaning and strong interpretability [6]. Guo et al. proposed a dynamically constrained physics-informed neural network (DcPINN), which effectively addressed the modeling and diagnosis challenges of complex mechanical systems with missing historical samples by incorporating physical boundary equations into a data-driven model [7]. Feng et al. developed a hybrid prediction model that combines an unscented Kalman filter (UKF) with deep learning, successfully overcoming the severe prediction errors caused by strong system nonlinearity in purely physics-based models under complex operating conditions [8]. Wang et al. constructed an analytical dynamic mechanism model of a mechanical rotor system containing local defects and misalignment errors, and by rigorously solving the internal multi-degree-of-freedom nonlinear differential equations of the system, they solved the difficulty of conducting purely theoretical quantitative derivation and precise quantification of the equipment’s micro-vibration responses under complex coupled faults [9]. Li et al. proposed a novel wavelet-constrained physics-informed neural network, which employed wavelet techniques to constrain the physical training direction of the model and thereby resolved the limited adaptability of a single model in dynamic scenarios involving cross-device and varying operating conditions [10]. However, such methods often rely on precise system mechanism equations and therefore struggle to address the highly nonlinear, multi-source heterogeneous, and data-missing uncertain environments commonly encountered in industrial settings, resulting in insufficient generalization capability under dynamically changing conditions.

In response to this need, data–mechanism hybrid-driven models have emerged, aiming to combine the fitting advantages of data-driven approaches with the interpretability of mechanism-based models. Among them, the Belief Rule Base (BRB), as a representative hybrid-driven framework, has become an ideal solution to the above challenges because it can organically integrate expert knowledge and observational data within an interpretable rule structure while effectively handling various forms of uncertainty [11]. Lian et al. proposed a BRB model with an adaptive nonlinear membership function, which addressed the degradation in prediction accuracy caused by the non-uniform distribution of quantitative observational data in complex equipment fault diagnosis [12]. Yin et al. developed an interpretable health and safety assessment model with dual-metric balance, effectively overcoming the difficulty that conventional assessment models tend to lose internal transparency and expert interpretability when high-accuracy parameter optimization is pursued [13]. Song et al. constructed a security situation assessment framework for industrial control systems based on evidential reasoning and BRB, successfully addressing the assessment bias caused by semiquantitative industrial field data and multisource uncertainty [14]. For feature prediction in complex systems, Kabir et al. proposed an integrated prediction framework combining deep learning and BRB, which exploited the robustness of expert prior knowledge to filter and correct anomalous noise, thereby resolving the tendency of purely data-driven models to fail when faced with uncertain sensor data streams [15]. At present, BRBs have been widely applied to tasks such as fault diagnosis [16,17,18,19,20], safety assessment [21], and industrial process prediction [22,23].

However, BRB models still suffer from significant limitations in practical deployment. On the one hand, the expansion of the rule base—driven by increasing input attributes and referential values—often leads to rule redundancy and the “curse of dimensionality.” This substantially increases optimization difficulty and computational overhead [24]. To mitigate these issues, recent studies have explored structural strategies such as compact BRB learning, hierarchical architectures, and rule reduction [11,25,26]. Nevertheless, these methods primarily focus on scaling down the model size, often overlooking the systematic prediction bias introduced by such structural simplifications [27]. This residual error, which has long been neglected, directly constrains the upper limit of predictive accuracy, making it difficult to satisfy the high-reliability requirements of industrial-grade applications [28].

On the other hand, a critical trade-off exists between predictive precision and model interpretability [13]. Aggressive parameter optimization or radical rule reduction can compromise the semantic consistency of the original rule base. Consequently, achieving a synergistic balance between structural compactness, optimization efficiency, and high-fidelity accuracy, while preserving rule-centric interpretability, remains a formidable challenge in BRB-based industrial forecasting.

Therefore, in this paper, a belief rule base model with adaptive rule reduction based on sensitivity analysis is proposed. The main contributions of this study are as follows.

(1) An MSE-convergence-fluctuation-driven rule sensitivity analysis mechanism is proposed to adaptively determine the rule screening threshold according to the convergence characteristics of the optimization process, thereby avoiding the instability and subjective cognitive bias caused by traditional empirically specified thresholds.
(2) A reduced-dimensional parameter optimization framework is constructed for the retained core rule subset. Under the premise of preserving the main BRB inference structure, the proposed framework effectively reduces the parameter search space and improves model compactness and optimization efficiency.
(3) A posterior residual consistency calibration module is introduced to compensate for the systematic prediction bias caused by structural simplification. Without modifying the rule structure, rule activation process, or evidential reasoning mechanism of the core BRB model, the proposed module further improves prediction accuracy.

The interpretability of the proposed framework mainly originates from the BRB-based reasoning structure, while the posterior calibration module only serves as an auxiliary residual compensation mechanism operating in the output space.

The remainder of this paper is organized as follows. Section 2 provides an in-depth analysis of the key challenges encountered in existing modeling processes and presents the corresponding solution strategies. Section 3 systematically describes the construction framework of the proposed model and its core algorithmic procedure. Section 4 comprehensively validates the effectiveness and robustness of the proposed model through comparative experiments based on a real-world oil pipeline leakage dataset. Finally, Section 5 summarizes the main contributions of this work and discusses directions for future research.

## 2. Problem Description

Problem 1: As the number of referential values in a BRB model increases, rule explosion is likely to occur. How can redundant rules be adaptively identified and removed while avoiding the instability caused by empirically specified thresholds, thereby reducing model complexity?

During the optimization process of a BRB model, a full-parameter optimization strategy is typically adopted, in which antecedent attributes, belief degrees, and rule weights are globally adjusted to improve prediction performance. However, as the number of referential values increases, the number of rules often grows exponentially, resulting in an increasingly bloated model structure as well as severe rule redundancy and high-dimensional parameter space problems.

Existing rule reduction methods mainly rely on empirically specified fixed thresholds to identify redundant rules. However, fixed thresholds often lack adaptability under different industrial datasets and operating conditions, which may lead to excessive rule elimination. Therefore, how to adaptively identify effective rules and reasonably determine a stable and reliable rule reduction threshold has become an important issue in BRB model optimization.

To address the above problem, this paper proposes an adaptive rule sensitivity analysis mechanism based on MSE convergence fluctuations. By analyzing the stable convergence characteristics during the optimization process, the contribution of each rule to the model output can be quantitatively evaluated, thereby enabling adaptive rule screening and effective compression of the rule base structure.

Problem 2: After rule reduction is completed, how can the retained core rule subset be effectively re-optimized in a reduced-dimensional parameter space to improve optimization efficiency while maintaining model performance?

Although rule reduction can effectively compress the model structure, directly removing rules may destroy the original cooperative relationships among parameters in the BRB model. If the reduced model is directly used for inference without further adjustment, prediction performance may deteriorate because of the structural imbalance introduced by rule removal.

To address this issue, the original optimization process is decomposed into two successive stages, thereby forming a staged optimization framework. After adaptive rule reduction is completed, the optimization parameter set is reconstructed according to the retained effective rules, and the subsequent optimization process is confined to a reduced-dimensional parameter space for refined optimization. This strategy fully exploits the computational advantages brought by parameter dimensionality reduction, improves optimization efficiency, and further adjusts the retained parameters to maintain prediction performance while reducing overall model complexity and overfitting risk.

Problem 3: How can predictive accuracy be maintained when structural simplification leads to information loss in the model?

Although rule compression can effectively improve model efficiency and reduce complexity, it inevitably causes partial information loss and introduces structural approximation errors, resulting in systematic residual bias in the output of the reduced BRB. To address this issue, this paper introduces a posterior residual consistency calibration mechanism to compensate for such errors without altering the core inference structure of the BRB. In the compensation process, the deviation between the true output of the training data and the prediction generated by the main BRB model is defined as the posterior residual. A residual learner is then constructed by jointly utilizing the input features and the BRB output to perform consistency calibration on the predictions of the main model. This mechanism does not modify the internal rule base, activation weights, or evidence fusion process of the BRB; instead, it models the remaining systematic error only in the output space. Therefore, it can be regarded as a post-optimization calibration framework designed for structural approximation bias.

## 3. Construction Procedure of the BRB-ARR Model

This section is organized as follows. Section 3.1 provides an overview of the overall architecture of the BRB-ARR model. Section 3.2 supplements the foundational knowledge for BRB model construction. Section 3.3 details the adaptive sensitivity analysis-based rule reduction process of the BRB-ARR model. Section 3.4 introduces the parameter optimization procedure of the model. Section 3.5 further discusses the optimization process after parameter dimensionality reduction induced by rule reduction. Finally, Section 3.6 explains the operational mechanism of the posterior calibration module in the BRB-ARR model.

### 3.1. Overview of the BRB-ARR Architecture

**Figure 1:**
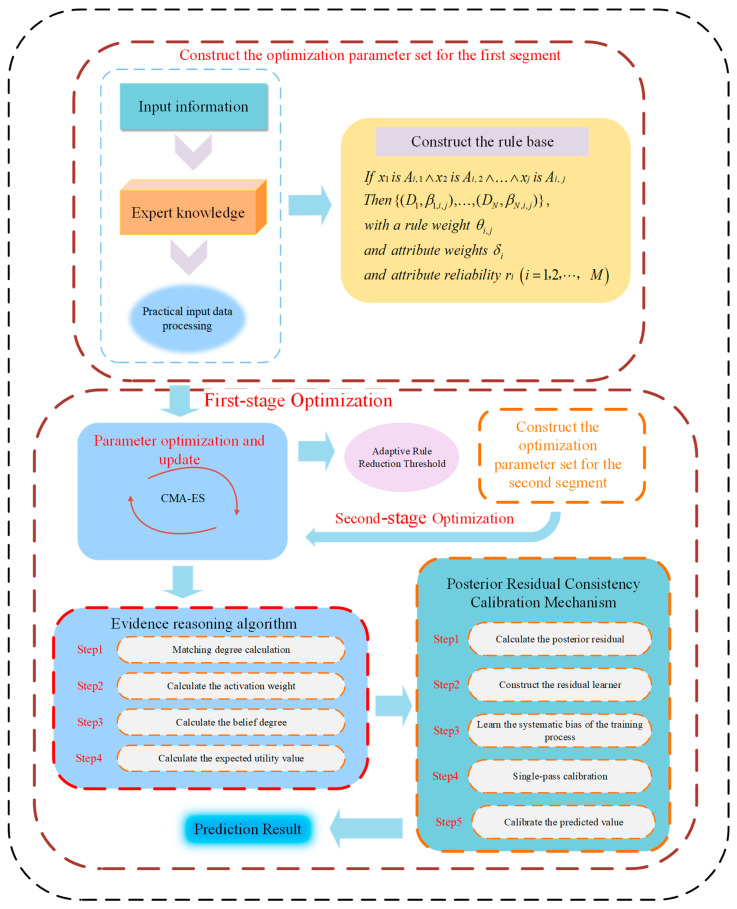
Overall Architecture of the BRB-ARR Model.

In complex nonlinear regression tasks for industrial safety, the BRB demonstrates distinct advantages owing to its strong ability to handle uncertainty and its transparent inference logic. However, the combined explosion of rules, together with the inevitable systematic bias introduced after structural reduction, severely constrains the predictive accuracy of the model in high-dimensional feature scenarios. To address this issue, this paper proposes a adaptively reduced BRB model that integrates adaptive sensitivity analysis with posterior residual calibration. This section systematically presents the complete inference mechanism and construction procedure of the proposed model. The specific structure is shown in Figure 1.

Step 1. The model adopts a two-stage optimization strategy to accomplish the overall decision-making process. First, a basic BRB model is constructed and optimized.

Step 2. In the first optimization stage, all the model parameters are globally optimized to obtain a benchmark model with satisfactory performance.

Step 3. When the training process in the later period of the first optimization stage becomes stable, sensitivity analysis is performed to evaluate the contribution or sensitivity of each rule in the benchmark model to the output, thereby adaptively determining the rule screening threshold.

Step 4. On the basis of the adaptive threshold, effective rules that make substantial contributions to model performance are retained, whereas redundant and low-efficiency rules in the rule base are eliminated.

Step 5. Based on the selected effective rules, a low-dimensional parameter space is constructed, and the model is further optimized in this subspace during the second stage, thereby reducing model complexity while preserving predictive performance.

Step 6. To address the systematic bias that may be introduced during model simplification, a postprocessing residual calibration module is incorporated to correct the output of the optimized model, thereby yielding the final prediction results while preserving model interpretability.

### 3.2. Basic Knowledge of the BRB Model

BRB models achieve reliable state assessment by mapping expert prior knowledge to multi-source input features. Within the BRB framework, the knowledge base consists of a series of IF–THEN rules that characterize the nonlinear mapping relationship between inputs and outputs. The system comprises M antecedent attribute input variables, denoted as $U_{1},U_{2},\ldots ,U_{M}$, and N referential grades of assessment outcomes, denoted as $D_{1},D_{2},\ldots ,D_{N}$. The internal structure of the k‐th rule can be defined as shown in Equation (1).

(1) $$\begin{array}{l} R_{k}:\mathrm{IF}\;U_{1}\;\mathrm{is}\;A_{k,1}\wedge U_{2}\;\mathrm{is}\;A_{k,2},\ldots ,\wedge U_{M}\;\mathrm{is}\;A_{k,M}\;\\ \mathrm{THEN}\;\{(D_{1},\beta_{k,1}),\ldots ,(D_{N},\beta_{k,N})\} \end{array}$$

where $A_{k,m}$ denotes the semantic referential value corresponding to the m‐th input feature in the k‐th rule; $\beta_{k,j}$ represents the belief degree assigned to the j‐th evaluation grade $D_{j}$ when the rule is activated. To quantify the relative importance of different features and rules within the system, $\theta_{k}$ denotes the global weight of the k‐th rule, which is used to measure the reliability of this rule in the entire knowledge base; $\delta_{m}$ denotes the importance coefficient of the m‐th antecedent attribute, which reflects the contribution intensity of this feature to the final decision outcome. All of the above weights and belief degree parameters are strictly constrained within the real-valued interval from zero to one.

First, since real industrial observational data are generally continuous-valued, they need to be transformed into activation distributions corresponding to the referential values in the rule base. For the actual observed value $x_{m}$ of the m‐th input feature, the attribute space to which it belongs is partitioned into $J_{m}$ discrete referential nodes, denoted as $\left\{A_{m,1},A_{m,2},\ldots ,A_{m,J_{m}}\right\}$. When the observed value $x_{m}$ falls between two adjacent referential nodes, $A_{m,i}$ and $A_{m,i+1}$, the specific transformation process is given in Equations (2) and (3).

(2) $$\alpha_{m,i}=\frac{A_{m,i+1}-x_{m}}{A_{m,i+1}-A_{m,i}}$$

(3) $$\alpha_{m,i+1}=\frac{x_{m}-A_{m,i}}{A_{m,i+1}-A_{m,i}}$$

where the matching degree of the m‐th attribute in the k‐th rule is denoted by $\alpha_{k,m}=\alpha_{m,i}$.

After the matching degree of each individual attribute is obtained, the model calculates the combined activation weight of each rule. The actual activation weight of the k‐th rule, denoted by $w_{k}$, is computed by aggregating the matching degrees of the rule over all the attributes, the attribute importance coefficient $\delta_{m}$, and the rule’s own relative weight $\theta_{k}$. The corresponding nonlinear calculation mechanism is given in Equation (4).

(4) $$w_{k}=\frac{\theta_{k}\prod_{m=1}^{M}{\left(\alpha_{k,m}\right)}^{\delta_{m}}}{\sum_{i=1}^{L}\left[\theta_{i}\prod_{m=1}^{M}{\left(\alpha_{i,m}\right)}^{\delta_{m}}\right]}$$

Finally, after the normalized activation weights of the rules are obtained, the model employs an analytical evidential reasoning algorithm to perform nonlinear aggregation of multiple independently activated rules. By combining the activation weight $w_{k}$ and the rule belief degree $\beta_{k,j}$, the system generates the final global output belief degree for each grade, denoted by $\beta_{j}$. The corresponding aggregation process is presented in Equations (5) and (6).

(5) $$\beta_{j}=\frac{\mu \left[\prod_{k=1}^{L}\left(w_{k}\beta_{k,j}+1-w_{k}\sum_{i=1}^{N}\beta_{k,i}\right)-\prod_{k=1}^{L}\left(1-w_{k}\sum_{i=1}^{N}\beta_{k,i}\right)\right]}{1-\mu \prod_{k=1}^{L}\left(1-w_{k}\right)}$$

(6) $$\mu ={\left[\sum_{j=1}^{N}\prod_{k=1}^{L}\left(w_{k}\beta_{k,j}+1-w_{k}\sum_{i=1}^{N}\beta_{k,i})-(N-1\right)\prod_{k=1}^{L}\left(1-w_{k}\sum_{i=1}^{N}\beta_{k,i}\right)\right]}^{-1}$$

μ denotes the orthonormalization correction factor. The final predicted output $y_{brb}$ of the complex industrial system is calculated as the expected weighted sum of the assessment reference grades $D_{j}$ and their corresponding output belief degrees $\beta_{j}$.

(7) $$y_{brb}=\sum_{j=1}^{N}D_{j}\beta_{j}$$

### 3.3. Adaptive Rule Reduction Driven by MSE Convergence Fluctuations

After the benchmark parameter optimization of the belief rule base has been completed, the model acquires a preliminary global fitting capability. However, as the number of referential values for the attributes increases, the rule base is highly susceptible to the curse of dimensionality, leading to a large number of redundant rules that contribute only marginally to the predictive output. To achieve structural compactness without compromising the physical interpretability of the model, this paper proposes an adaptive rule reduction mechanism. Specifically, this mechanism evaluates the local sensitivity of each individual rule and generates a screening threshold by incorporating the convergence fluctuation characteristics observed in the late stage of optimization, thereby enabling the precise elimination of ineffective rules.

The sensitivity of a rule objectively reflects its core significance within the entire inference and decision-making network. To quantify the specific contribution of each rule, this paper proposes an adaptive sensitivity analysis mechanism. Let the globally optimal parameter vector be denoted by $\Theta_{best}$, and let the baseline mean squared error of the system under this parameter setting be defined as $E_{base}=E(\Theta_{best})$. For the k‐th rule in the rule base, the system constructs a locally perturbed parameter vector $\Theta_{\setminus k}$ by masking this rule. The perturbed vector is then reintroduced into the model for forward evidential inference, yielding a new system error $E_{sa,k}=E(\Theta_{\setminus k})$. The absolute sensitivity contribution of the k‐th rule, denoted by $g_{k}$, is rigorously defined as the absolute value of the error variation after this rule is removed. Its mathematical formulation is given in Equation (8).

(8) $$g_{k}=|E_{sa,k}-E_{base}|$$

By calculating the sensitivity contributions of all L rules, the system obtains a global sensitivity distribution vector that characterizes the importance gradient of the rules.

Conventional rule reduction methods largely rely on fixed empirical constants as truncation thresholds, which can easily lead to either excessive reduction or redundant rule retention when applied to different industrial datasets. To improve the robustness of the algorithm, the proposed model is used to construct an adaptive threshold by exploiting the fluctuation characteristics of the objective function during the late stage of optimization convergence. In the final stage of parameter optimization, the model extracts the mean squared error sequence from the last $G_{tail}$ iterations. It then computes the set of absolute error variations between adjacent iterations and takes the statistical median of its nonzero elements to quantify the intrinsic search noise scale under the current data distribution, denoted by the relative noise coefficient $\rho_{noise}$. The determination of the adaptive rule screening threshold $T_{sa}$ comprehensively considers the historical fluctuation-driven term, the lower bound of the baseline error proportion, and the absolute anti-degeneration minimum, and its construction process is given in Equation (9).

(9) $$T_{sa}=\max\left(\lambda \cdot \rho_{noise}\cdot E_{base},\;\gamma \cdot E_{base},\;\epsilon_{min}\right)$$

λ denotes the fluctuation amplification adjustment coefficient, which is primarily used to smooth random noise spikes; γ is the factor for strengthening the lower-bound proportion, ensuring that the threshold does not shrink abnormally when the baseline error is relatively large; and $\epsilon_{min}$ is the minimum truncation constant introduced to prevent threshold invalid penetration during the high-precision convergence stage.

On the basis of the adaptive threshold $T_{sa}$, deterministic pruning and reconstruction are performed on the original knowledge base. The absolute sensitivity $g_{k}$ of each rule is then subjected to the following criterion:

(10) $$I_{k}=\left\{\begin{array}{ll} 1, & \mathrm{if}\;g_{k}\geq T_{sa}\\ 0, & \mathrm{if}\;g_{k}<T_{sa} \end{array}\right.$$

where the state indicator variable $I_{k}$ denotes whether a rule is ultimately retained. For a rule with $I_{k}=1$, the system identifies it as a strong decision node with significant information gain and incorporates it into the core rule subset for preservation. A rule with $I_{k}=0$ is regarded as an invalid computational unit and is permanently removed from the original model structure. Through this process, the rule dimension is substantially compressed in a purely data-driven manner, thereby establishing a solid foundation for the subsequent secondary optimization in a low-dimensional space.

### 3.4. Parameter Optimization

**Figure 2:**
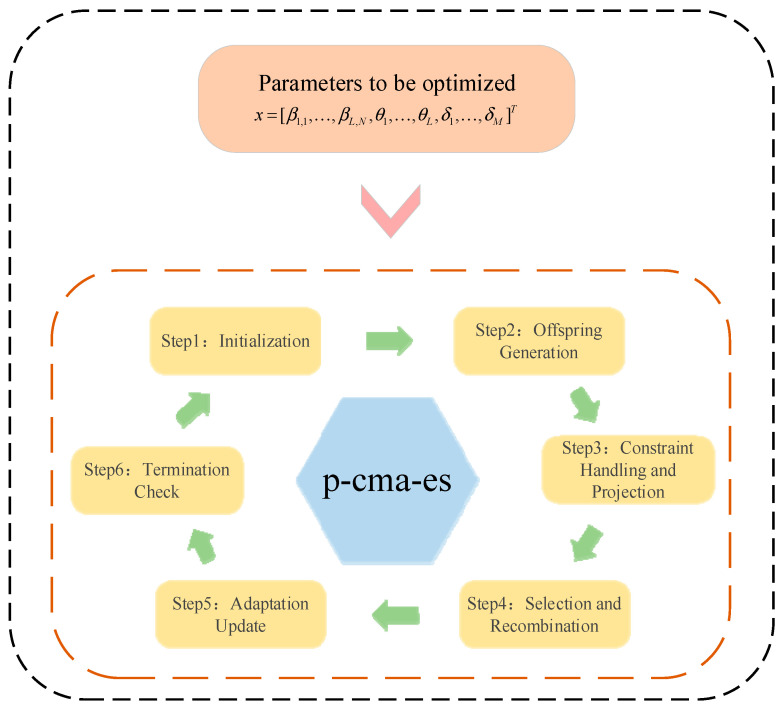
Flowchart of the Proposed Optimization Algorithm.

To improve the predictive accuracy and reliability of the BRB-ARR model, this paper employs a projection-based covariance matrix adaptation evolution strategy (P-CMA-ES) to perform global optimization of the core internal parameters of the model. The complete optimization problem formulation and the corresponding algorithmic evolution procedure are presented below, as illustrated in Figure 2.

Figure 2 illustrates the overall optimization framework of the proposed P-CMA-ES-based BRB-ARR parameter optimization process. The optimization procedure first performs parameter initialization. Subsequently, offspring individuals are sampled during the optimization process to generate candidate solutions. To satisfy the physical constraints and completeness requirements of the BRB-ARR model, the generated candidate solutions are further subjected to boundary truncation and orthogonal projection correction. Afterward, the candidate solutions are evaluated according to the model fitness, and the parameter distribution is updated through elite selection and weighted recombination. Finally, the covariance matrix, evolution paths, and global step size are adaptively updated to guide the algorithm toward the optimal solution progressively. The optimization process is terminated when the predefined convergence criterion is satisfied or the maximum number of iterations is reached.

The set of parameters to be optimized in the BRB-ARR model is given as follows.

(11) $$x={\left[\beta_{1,1},\ldots ,\beta_{L,N},\theta_{1},\ldots ,\theta_{L},\delta_{1},\ldots ,\delta_{M}\right]}^{T}$$

The optimization objective of the model is to minimize the MSE between the predicted output $\hat{y}$ and the true training label y. Moreover, the optimization process must satisfy the completeness axiom of the BRB-ARR model as well as the physical constraints imposed on the parameters. The optimization model can therefore be formulated as follows.

(12) $$\begin{array}{l} \min f(x)=\frac{1}{T}\sum_{t=1}^{T}{\left(y_{t}-{\hat{y}}_{t}\right)}^{2}\\ s.t.\;\sum_{n=1}^{N}\beta_{k,n}=1,\;k=1,2,\ldots ,L\\ 0\leq \beta_{k,n}\leq 1,\;0\leq \theta_{k}\leq 1,\;0\leq \delta_{m}\leq 1 \end{array}$$

T denotes the total number of training samples.

For the above nonlinear optimization problem with strict equality and inequality constraints, this paper adopts a covariance matrix adaptation algorithm embedded with a projection operator. The specific iterative procedure is described as follows.

Step 1: Parameter Initialization

Initialize the population size as λ=10+3lnD, and set the number of selected parent solutions to μ=λ/2. The recombination weights $w_{i}$ are computed and normalized.

Afterward, initialize the mean vector $m^{\left(0\right)}$, the global initial step size $\sigma^{\left(0\right)}$, the covariance matrix $C^{\left(0\right)}=I$, and the evolution paths $p_{c}=0$ and $p_{\sigma }=0$.

Step 2: Population Sampling

In the g‐th generation, λ candidate solutions are generated by sampling from a multivariate normal distribution based on the current mean vector $m^{\left(g\right)}$ and covariance matrix $C^{\left(g\right)}$.

(13) $$x_{i}^{\left(g+1\right)}\sim m^{\left(g\right)}+\sigma^{\left(g\right)}N(0,C^{\left(g\right)}),\;i=1,\ldots ,\lambda$$

Step 3: Constraint Handling and Projection Correction

The generated candidate solutions are first subjected to boundary truncation to ensure that all the parameters are within the interval [0,1]. With respect to the equality constraint of the sum of belief degrees $\sum \beta_{k,n}=1$, orthogonal projection correction is performed on the belief-degree block in the decision vector.

(14) $$x_{sub}=x_{sub}-A^{T}{\left(AA^{T}\right)}^{-1}(Ax_{sub}-1)$$

Step 4: Selection and Recombination

The corrected candidate solution $x_{i}$ is mapped back to the BRB parameters, and the fitness value $f(x_{i})$ of the model under this solution is evaluated and recorded. The λ candidate solutions are then ranked in ascending order according to their fitness values. The top μ individuals with the best performance are selected, and the mean of the parameter distribution for the next generation is updated by taking their weighted sum using the logarithmic weights $w_{i}$.

(15) $$m^{\left(g+1\right)}=\sum_{i=1}^{\mu }w_{i}x_{i:\lambda }^{\left(g+1\right)}$$

Step 5: Adaptive Parameter Update

The internal strategy parameters of the algorithm are then updated. First, the conjugate evolution path $p_{\sigma }$ is computed, and the global step size $\sigma^{\left(g+1\right)}$ is updated by comparing its norm with the expected random norm. Subsequently, the covariance evolution path $p_{c}$ is updated. By combining the rank-one update based on $p_{c}$ with the rank μ update derived from the top μ elite individuals in the current generation, the covariance matrix $C^{\left(g+1\right)}$ is adaptively adjusted. In addition, eigen decomposition of $C^{\left(g+1\right)}$ is performed periodically to update the orthogonal coordinate system.

Step 6: Check the termination condition

The algorithm checks whether the current number of function evaluations has reached the preset maximum iteration limit or whether the best fitness value in the population has fallen below the minimum convergence threshold. If either condition is satisfied, the iterative process is terminated, and the historically optimal parameter combination is returned. Otherwise, let g=g+1, and return to Step 2 to continue the evolutionary process.

### 3.5. Reduced-Dimensional Parameter Optimization Mechanism

After redundant rules in the rule base have been removed, the model successfully eliminates ineffective knowledge units. However, directly removing certain rules disrupts the original cooperative balance among the globally optimal parameters. If inference is performed directly on the basis of the reduced parameter system, the nonlinear mapping relationship of the model may become distorted, thereby leading to a significant decline in predictive accuracy. To address this issue, this paper proposes a secondary optimization mechanism based on a reduced-dimensional parameter space.

Let $L^{\prime }$ denote the number of retained core effective rules after adaptive screening. On the basis of this subset of effective rules, the model reconstructs the original high-dimensional decision space in a reduced-dimensional manner, thereby yielding a more compact low-dimensional parameter vector $\Theta_{sub}$. Accordingly, the total dimensionality of the parameters to be optimized is significantly reduced from that of the original full-state space to $L^{\prime }\times N+L^{\prime }+M$.

The reconstructed low-dimensional parameter vector is subsequently treated as an independent optimization target, and the evolutionary strategy algorithm equipped with an adaptive covariance matrix adjustment mechanism is reinitialized under the strict constraints of probability normalization equations and non-negativity boundary conditions. The objective of this second-stage optimization remains the minimization of the global mean squared prediction error. Owing to the substantial reduction in the search dimensionality, the convergence efficiency of the optimization algorithm is markedly improved, enabling the rapid reestablishment of the collaborative response relationships among the retained rules. This mechanism effectively compensates for the approximation error introduced by structural compression of the model, thereby maintaining high fitting accuracy while ensuring structural compactness.

### 3.6. Posterior Consistency Calibration

After adaptive rule reduction and reduced-dimensional optimization, the rule base of the BRB-ARR model effectively improves structural compactness and computational efficiency. However, because the underlying inference process relies heavily on discretized mapping of referential values and rule reduction inevitably causes information loss in the feature space, the model may still retain a certain degree of local systematic bias when complex continuous nonlinear boundaries are characterized. To completely eliminate such structural approximation errors, this paper proposes a posterior residual consistency calibration mechanism while fully preserving the physical interpretability of the main model.

Notably, the proposed residual learning mechanism does not constitute a re-modeling of the original prediction task, nor does it replace the primary BRB-based decision-making framework. The belief rule base continues to perform the core feature inference task, while its internal rule structure, activation weights, and evidential reasoning process remain unchanged. Therefore, the semantic transparency and rule-level interpretability of the primary BRB inference architecture are preserved throughout the entire prediction process.

It should be noted that the posterior residual calibration module itself is not a rule-level interpretable model. Instead, it operates only as an auxiliary post-processing compensation mechanism in the output space. Its purpose is to model and compensate for the structural approximation bias that cannot be fully characterized by the reduced BRB structure. Since the residual learner does not modify the original rule semantics, inference logic, or evidential fusion mechanism, the interpretability of the core BRB reasoning process is not destroyed.

Consequently, the proposed BRB-ARR framework should be regarded as a rule-centered interpretable prediction framework with auxiliary posterior residual compensation, rather than a completely interpretable model in the strict sense.

In the specific mathematical modeling process, the system first performs an analytical decomposition in the residual error space. Let X denote the original multidimensional feature input matrix of the training set, and let the deterministic evaluation output of the main model after reduced-dimensional secondary optimization be denoted by $y_{brb}$. Moreover, let the corresponding true observed label of the sample be $y_{true}$. The system then rigorously defines the difference between the true target and the prediction of the main model as the posterior residual r. The corresponding computation is given in Equation (16).

(16) $$r=y_{true}-y_{brb}$$

After the residual target has been extracted, the system constructs a nonlinear compensation engine to fit the structural bias. To fully exploit the available inference information, the residual learner does not rely solely on the original feature channel; instead, it performs feature-level concatenation of the original input feature matrix X and the main model prediction $y_{brb}$, thereby forming an enhanced joint feature input matrix $X_{res}$.

In selecting the compensation algorithm, this paper adopts the least-squares gradient boosting tree as the residual calibrator. This algorithm is capable of flexibly and accurately fitting high-frequency and complex nonlinear residual distributions, making it particularly suitable for characterizing the systematic bias retained by the reduced model in different feature subspaces. Unlike directly employing a purely black-box deep learning network as the primary predictor, the compensation algorithm in this study operates only within the low-dimensional residual error space, with both the model scale and fitting depth being strictly controlled. Therefore, as a lightweight posterior calibration tool, this algorithm is highly compatible with the engineering requirements of the present study.

By establishing a nonlinear mapping from the joint feature matrix $X_{res}$ to the predicted residual $\hat{r}$ using the least-squares gradient boosting tree algorithm, the final consistency prediction value of the complex industrial system, denoted by $y_{final}$, is generated through a linear additive combination of the stable output of the primary decision-making model and the dynamically corrected bias produced by the calibrator. The corresponding synthesis process is given in Equation (17).

(17) $$y_{final}=y_{brb}+\hat{r}$$

To ensure the objectivity of the evaluation and prevent information leakage, the posterior calibration module operates under a strict isolation protocol. The parameters of the residual learner are estimated exclusively using the training residuals $\hat{r}$ derived from the training dataset. During the testing phase, the residual learner remains frozen and processes only the independent testing samples to generate correction values. This ensures that the performance gains reported on the testing set reflect the true generalization ability of the model rather than over fitting to the evaluation data.

The above posterior consistency calibration mechanism achieves effective decoupling, in physical terms, between transparent inference and black-box compensation. The interpretability of expert knowledge at the primary decision-making level is fully preserved, while the overall predictive accuracy limit and anti-interference robustness of the model are significantly enhanced through precise error correction in the residual space.

## 4. Case Study

In the context of modern manufacturing, safety prediction and early risk warning for industrial systems have become important foundations for ensuring the stable operation of production processes [29]. Owing to the typical structural complexity of industrial systems, their condition monitoring and maintenance face numerous challenges. Especially under complex operating conditions or in harsh environments, once a system anomaly occurs, it not only leads to equipment damage and resource waste but also may trigger safety accidents or even environmental risks. Therefore, accurate prediction and safety assessments of the operating states of industrial systems are highly important for enhancing overall system reliability and safety.

To verify the effectiveness of the proposed BRB-ARR model in safety prediction for complex industrial systems, oil pipeline leakage data from a typical industrial scenario are selected as the experimental subject for analysis and validation. Specifically, Section 4.1 details the dataset used in the experiment, including data sources and feature composition; Section 4.2 elaborates on the construction process of the BRB-ARR model in oil pipeline leakage prediction; Section 4.3 validates and analyzes the predictive performance of the model on the basis of actual data; Section 4.4 validation of the model generalization ability; and Section 4.5 summarizes the experimental results and discusses the application effectiveness of the model.

### 4.1. Data Description

The oil pipeline leakage data used in the experiments originate from a large-scale pipeline construction project in the United Kingdom with a total length exceeding 100 km [30]. In this project, flow meters and pressure gauges were installed at the inlet and outlet of the pipeline, respectively. Simultaneously, eight intermediate monitoring points were established along the pipeline and equipped with corresponding pressure detection devices to record various data during the pipeline’s operation. Under normal operating conditions, the pipeline maintains stable operation. However, when the inlet flow and outlet flow are inconsistent, the internal pressure of the pipeline changes accordingly; a significant anomaly may indicate a risk of pipeline leakage. Therefore, the difference between the inlet and outlet flows (Flow Diff) and the variation in the average pipeline pressure over time (Press Diff) serve as two key indicators for identifying pipeline leakage. The dataset included 2008 samples during pipeline leakage events at a sampling frequency of one sample every 10 s.

### 4.2. Model Construction

In the construction of the BRB-ARR model, eight reference levels are defined for the flow difference attribute to characterize its state variations across different intensities: negative very large (NVL), negative large (NL), negative generally large (NGL), negative medium (NM), negative small (NS), negative very small (NVS), positive small (PS), and positive medium (PM). For the Press Diff attribute, seven reference levels are employed to describe its dynamic characteristics: negative large (NL), negative medium (NM), negative small (NS), very small (VS), small (S), positive medium (PM), and positive large (PL). The input reference values corresponding to these two input attributes, Flow Diff and Press Diff, are listed in Table 1.

In this paper, 400 iterations of BRB model optimization are used as a benchmark method for the comparative analysis of model performance and computational complexity. Building upon this, the BRB-ARR model introduces adaptive rule reduction and posterior residual verification mechanisms to construct an efficient and adaptive optimization framework. The BRB-ARR model adopts a two-stage optimization strategy: First, 200 iterations of initial optimization are executed to allow the model parameters to rapidly reach a preliminary convergence state with low computational overhead; subsequently, after the optimization process enters a stable phase, an adaptive rule reduction mechanism based on sensitivity analysis is introduced. This mechanism adaptively determines a reduction threshold according to the contribution of each rule to the model output, thereby filtering out redundant or low-contribution rules to achieve the streamlining and restructuring of the rule base.

After the rule reduction is complete, the model undergoes a further 200 iterations of optimization within the streamlined rule space to enhance the specificity and efficiency of parameter learning. This collaborative “optimizing-reducing-reoptimizing” mechanism significantly reduces model complexity while effectively maintaining or even improving prediction accuracy.

In the process of constructing an adaptive threshold based on historical MSE convergence behavior during rule reduction, a fluctuation amplification coefficient λ is introduced to adjust the impact degree of error fluctuations in the tail section of convergence on the discrimination of rule sensitivity. Theoretically, the MSE variations in the optimization algorithm in the late convergence stage can be viewed as small random perturbations around the optimal solution, and their fluctuation amplitude reflects the noise level of the model in a stable state. Therefore, the relative fluctuation of the tail section is taken as the basis for threshold construction, and scale adjustment is performed through the coefficient λ. An appropriate λ can make the threshold both higher than the range of numerical perturbations and lower than the true impact of key rules on model performance, thereby achieving an effective distinction between valid and redundant rules.

**Figure 3:**
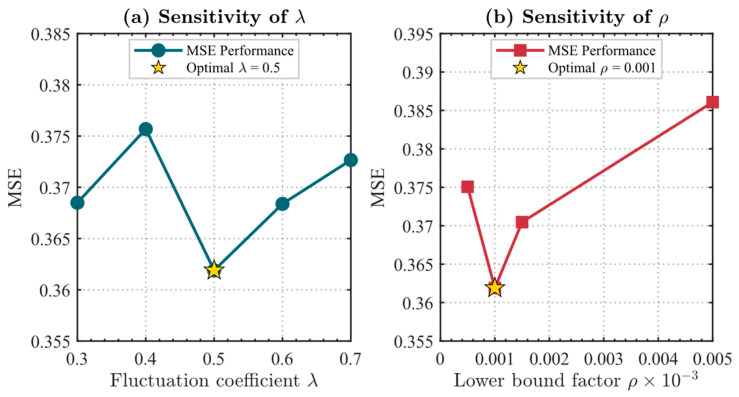
Sensitivity analysis experiment.

The primary role of the fluctuation amplification coefficient λ is to regulate the contribution of optimization tail noise to threshold generation. As illustrated in Figure 3a, the mean squared error of the model exhibits high stability when λ varies within the empirical range of 0.3 to 0.7. The model achieves the minimum mean squared error of 0.3619 in the petroleum pipeline case when λ is set to 0.5. This indicates that this value effectively captures the intrinsic fluctuation characteristics of the optimization process, avoiding both the retention of redundant rules due to a low threshold and the accidental removal of critical information due to an excessive threshold.

The lower bound proportion factor ρ defines the minimum contribution limit of a rule to the model output. As shown in Figure 3b, the performance was tested with ρ ranging from 0.0005 to 0.005. Experimental data indicate that setting ρ to 0.001 achieves the optimal balance between structural compression and predictive accuracy. This value aligns with the common one thousandth tolerance standard in industrial engineering modeling. When ρ is too large, the number of retained rules drops significantly to 12.7, leading to information loss and increased errors. Conversely, a value that is too small weakens the rule reduction effect. Therefore, selecting λ=0.5 and ρ=0.001 as baseline parameters is well supported by experimental evidence and logical rationality.

In the following, λ=0.5 is uniformly adopted. Theoretically, this value corresponds to a half-amplitude amplification of the fluctuations in the tail section of convergence, which can better characterize the typical perturbation scale in the stable phase while avoiding discrimination distortion caused by excessive amplification. At the experimental level, two independent experiments adopted the same parameter settings. The results show that the models both obtained a smaller MSE when and maintained stable convergence characteristics, indicating that this value has good applicability and robustness across different data scenarios.

Furthermore, when the value of λ is too small, the threshold tends to misjudge rules that still contribute to the model but have a weaker impact as redundant, leading to excessive reduction and affecting the model’s generalization capability; conversely, when λ is too large, the threshold is significantly raised, and a large number of low-contribution rules will be retained, weakening the rule reduction effect. Therefore, λ=0.5 achieves a good balance between suppressing noise interference and maintaining structural simplification, making the rule screening process robust while considering model performance.

It should be noted that the setting of λ is primarily based on empirical analysis of optimization convergence characteristics, and its role is to provide a stable and robust threshold scale, rather than being a precisely tuned parameter.

Furthermore, to further enhance the reliability and generalizability of the model, this paper proposes a posterior residual verification mechanism by incorporating residual distribution characteristics during the training process. While preserving the interpretability of the initial model, the mechanism performs consistency testing on the prediction results by combining model residuals with optimized parameter information, thereby achieving further evaluation and correction of the model output credibility.

### 4.3. Experimental Validation

The experiments in this study adopt the data partition commonly used in previous BRB-based oil pipeline leakage studies, where 500 samples are selected for model parameter training, and the testing data are used for final performance evaluation. The main BRB model, adaptive rule reduction process, reduced-dimensional parameter re-optimization, and posterior residual calibration module are all constructed and optimized only based on the training data. The testing samples are strictly isolated throughout the entire training and parameter tuning process, including the rule screening and residual learner construction stages. These unseen samples are introduced only in the final evaluation stage to verify the consistency and reliability of the calibrated prediction results.

To systematically evaluate the predictive performance of the proposed BRB-ARR model, an experimental validation is conducted on the basis of the aforementioned oil pipeline leakage dataset. The experiments focus primarily on comparing the prediction accuracy, convergence efficiency, and model complexity between the initial BRB model and the BRB-ARR model. First, a direct comparison of the prediction results is presented to intuitively demonstrate the overall fitting capability of the models.

**Figure 4:**
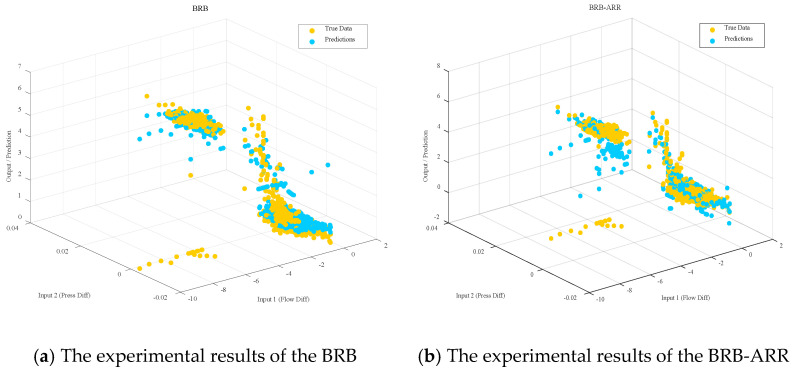
Comparison of the results between the BRB and BRB-ARR models.

Figure 4 presents a 3D scatter comparison between the true outputs and predicted outputs of the two models. Figure 4a shows the prediction results of the initial BRB model, while Figure 4b shows the prediction results of the proposed BRB-ARR model. In both subfigures, the x-axis and y-axis represent the two input attributes, namely Flow Diff and Press Diff, respectively, and the z-axis represents the output value. The yellow points denote the true data, whereas the blue points denote the predicted results. Compared with the initial BRB model, the predicted points of the BRB-ARR model are more closely distributed around the true data points, indicating that the proposed model achieves better fitting performance and prediction consistency.

Table 2 details the comparison results of the two models across various quantitative evaluation metrics. The data indicate that the BRB-ARR model achieves a comprehensive improvement in prediction accuracy. Its mean squared error (MSE), root mean square error (RMSE), and mean absolute error (MAE) decreased to 0.3619, 0.6015, and 0.2231, respectively, which are significantly lower than those of the initial BRB model (0.5291, 0.7274, and 0.3351, respectively). Moreover, the coefficient of determination ($R^{2}$) and variance accounted for (VAF) of the BRB-ARR model increased to 0.9386 and 93.91%, respectively, indicating that the model’s ability to explain data variance is further enhanced.

**Figure 5:**
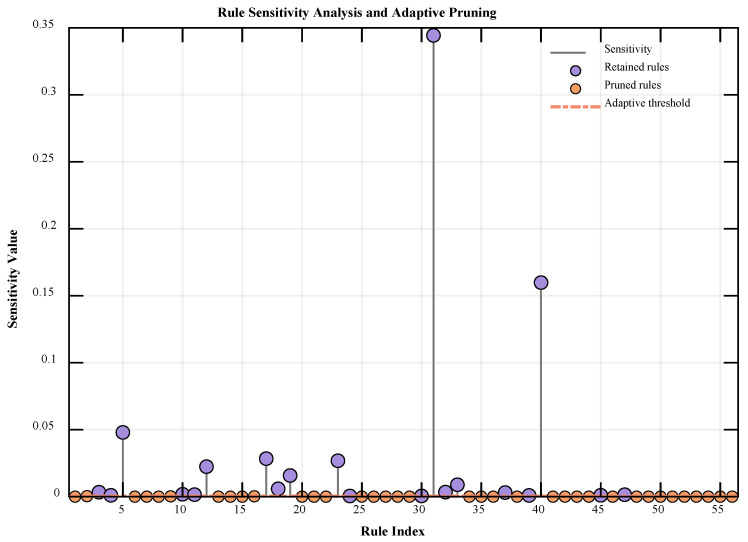
Adaptive sensitivity analysis rule reduction process.

Furthermore, Table 3 shows the standard deviation (Std) of each metric for the models after multiple experiments. The results show that the standard deviations of the BRB-ARR model across key error metrics remain low. This finding indicates that while significantly improving prediction accuracy, the BRB-ARR model still maintains good robustness and optimization stability. During the initial 200-round optimization process of the BRB-ARR model, an adaptive rule reduction mechanism based on sensitivity analysis is introduced in the later optimization stage. Figure 5 illustrates the sensitivity distribution of all rules during the rule screening process, where the x-axis represents the rule index and the y-axis represents the corresponding sensitivity value of each rule. The purple points denote the retained rules, while the orange points denote the pruned rules. The red dashed line represents the adaptively determined sensitivity threshold. By evaluating the contribution of each rule to the model output and comparing it with the adaptive threshold, redundant rules with low sensitivities can be effectively identified and eliminated, thereby achieving adaptive rule screening and structural refinement of the rule base.

Table 4 compares the training runtime of the BRB-ARR model and the original BRB model, further demonstrating the computational efficiency improvement brought by the proposed rule reduction mechanism.

To quantitatively analyze the computational efficiency, this study compares the training and inference times of the BRB-ARR model with the conventional BRB model as summarized in Table 4. The experimental results indicate that the training time of BRB-ARR is shortened by approximately 28.17 percent compared to the conventional model because the adaptive rule reduction mechanism eliminates a large number of redundant rules. More significantly, the inference time on the testing set decreases from 0.0274 s to 0.0103 s, representing a 62.43 percent improvement in computational efficiency. This improvement is directly attributed to the simplification of the model structure from 56 rules to approximately 20 rules and the reduction in parameter dimensionality from 338 to 122. The lower computational overhead demonstrates the application potential of this framework in real time industrial monitoring and high frequency decision making scenarios, which can satisfy the strict requirements of industrial grade deployment for response speed.

The experimental results indicate that the sensitivity values of most rules are close to zero, with only a few rules having a significant effect on the model output, suggesting a certain degree of redundancy in the original rule base. Under the adaptive rule reduction mechanism, the model retains only the key rules with high sensitivity, thereby achieving effective streamlining of the rule structure. Experimental statistics show that the average number of rules retained after reduction is approximately 20, representing a significant reduction in scale compared with the unreduced BRB model, with a reduction ratio exceeding 60%. Furthermore, the rule reduction process does not simply rely on a fixed threshold; instead, it adaptively determines the threshold on the basis of the sensitivity distribution in the stable state of the model during the late optimization stage, making the rule screening process more rational and data driven. This method effectively reduces model complexity while ensuring the complete preservation of key rule information, providing a more compact search space for subsequent parameter optimization.

Because the parameter optimization process of the BRB-ARR model involves a certain degree of stochastic search, slight variations may occur in the rule sensitivity distribution and the retained rule subset across different independent experiments. Therefore, the statement that the number of rules is reduced from 56 to approximately 20 does not mean that exactly 20 fixed rules are retained in a single experiment. Instead, it refers to the average statistical result obtained from 10 independent experiments. To further clarify the physical interpretability of the reduced rule base, the retention frequency of each rule was calculated over the 10 repeated experiments, and the frequently retained rules together with their corresponding physical meanings are summarized in Table 5.

As shown in Table 5, the frequently retained rules are mainly distributed in physically meaningful regions related to leakage states, leakage-boundary conditions, pressure-transition states, and normal operating transitions. Specifically, rules with negative FlowDiff and decreasing or nearly stable PressDiff generally reflect a state in which the outlet flow is lower than the inlet flow, accompanied by variations in pipeline pressure. This is consistent with the mechanisms of flow loss and pressure disturbance during pipeline leakage. In addition, several rules with near-zero or slightly positive FlowDiff are also frequently retained, indicating that the proposed adaptive rule reduction mechanism preserves not only the key rules directly associated with leakage, but also rules that help characterize boundary conditions and normal transition states. This contributes to reducing false alarms and improving the prediction stability of boundary samples.

It should be noted that rule retention in this study is not determined solely by the semantic severity of leakage implied by a rule. Instead, it depends on the actual sensitivity contribution of each rule to the prediction error under the current data distribution and optimized parameter state. Therefore, when certain extreme rules are rarely activated in the samples, or when their effects have already been represented by neighboring rules, their local sensitivity may be relatively low, and they may be removed by the adaptive reduction mechanism. This demonstrates that the proposed rule reduction process does not simply reduce the number of rules. Rather, it statistically preserves key rules that make more stable contributions to model inference and prediction, thereby maintaining rule-level physical interpretability while reducing the complexity of the rule base.

**Figure 6:**
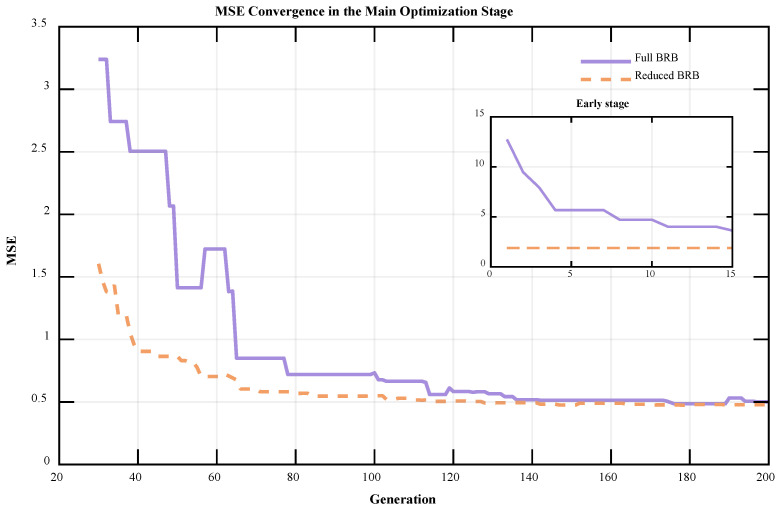
MSE convergence curves.

A comparison of the MSE convergence curves further verifies the effectiveness of the adaptive rule reduction mechanism in the BRB-ARR model. This mechanism effectively reduces the reasoning complexity of the model while ensuring that the reasoning accuracy of the original model remains largely unaffected, thereby improving optimization efficiency and model compactness. The MSE convergence curves of the initial BRB model and the BRB-ARR model during the final 200 iterations of the optimization process are shown in Figure 6. In this figure, the x-axis represents the generation number, and the y-axis represents the corresponding MSE value. The purple solid line denotes the convergence curve of the Full BRB model, while the orange dashed line denotes the convergence curve of the Reduced BRB model. The inset in the upper-right corner illustrates the convergence behavior during the early optimization stage, providing a clearer comparison of the convergence characteristics of the two models in the initial optimization process.

As shown by the MSE convergence curves in Figure 6, the BRB-ARR model exhibits superior convergence characteristics during the final 200 iterations of the optimization process. Compared with the original BRB model without rule reduction, the reduced BRB-ARR model achieves a lower overall MSE during the final 200-iteration optimization stage, and the convergence process is more stable. Especially in the initial stage of optimization, the BRB-ARR model can enter a lower error range more quickly, reflecting higher convergence efficiency. As the number of iterations increases, the MSEs of both models gradually decrease and tend to stabilize, but the BRB-ARR model consistently maintains a lower error level. This finding indicates that after rule reduction, the model does not sacrifice prediction accuracy while reducing complexity; instead, the optimization performance is improved to a certain extent. This finding demonstrates that the adaptive rule reduction mechanism effectively removes redundant rules and enhances the predictive ability of the model.

**Figure 7:**
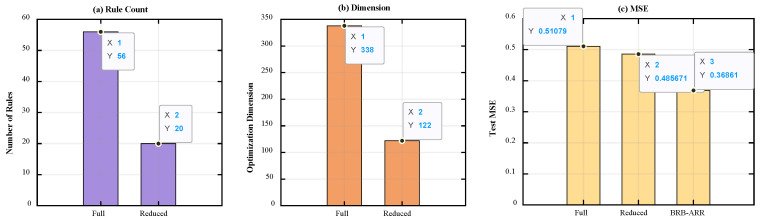
Changes in parameter dimensions and MSE after reduction.

**Figure 8:**
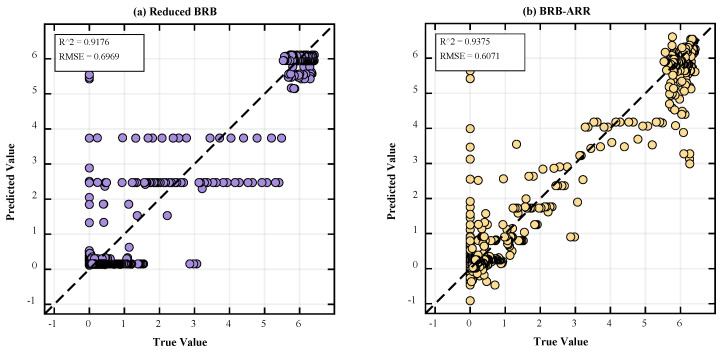
Prediction regression plot of the rule-reduced model incorporating. The posterior correction mechanism.

Comparisons of the parameter dimension changes before and after rule reduction are presented in Table 6 and Figure 7. Specifically, Figure 7a shows the comparison of rule numbers between the Full BRB model and the Reduced BRB model, while Figure 7b illustrates the corresponding optimization parameter dimensions before and after reduction. Figure 7c further compares the test MSE values of the Full BRB, Reduced BRB, and final BRB-ARR models. In these subfigures, the x-axis represents different model structures, and the y-axis represents the corresponding evaluation metric, including rule number, optimization dimension, or test MSE. The numerical labels above the bars denote the corresponding quantitative results of each model. Figure 8 presents the regression relationship comparison between the Reduced BRB model and the final BRB-ARR model. Figure 8a shows the prediction results of the Reduced BRB model, while Figure 8b shows the prediction results after posterior residual calibration. In both subfigures, the x-axis represents the true values, and the y-axis represents the predicted values. The dashed diagonal line denotes the ideal prediction line where the predicted values are equal to the true values. In addition, the corresponding R^2^ and RMSE values are provided in the upper-left corner of each subfigure to quantitatively evaluate the regression performance of the models.

Combining the results of Figure 7 and Table 6, it is evident that the model not only achieves rule reduction but also significantly reduces the optimization dimensionality from 338 to 122 as the number of rules decreases. This reduction in optimization dimensionality directly narrows the parameter search space, which helps improve training efficiency and optimization stability while reducing computational complexity. According to the average results of 10 experimental runs presented in Table 6, the BRB-ARR model achieves the best performance in terms of prediction accuracy. The test set MSE of the reduced model (Reduced BRB) is 0.4856, which does not lead to performance degradation compared with the original Full BRB model (MSE = 0.5291), but instead achieves a certain degree of performance improvement. This finding indicates that the rule reduction process helps enhance the generalization capability of the model after redundant information is removed. Furthermore, the test set MSE of the final BRB-ARR model is further reduced to 0.3619, demonstrating that the introduced posterior correction mechanism can effectively compensate for model residuals and further improve prediction accuracy.

As seen from the predicted scatter distribution in Figure 8, the reduced BRB-ARR model is already capable of fitting the actual values well in terms of the overall trend. After the posterior correction mechanism is incorporated, the consistency between the predicted results and the actual values is further enhanced, with the data points more closely concentrated around the diagonal. The corresponding evaluation metrics also indicate that the performance of the BRB-ARR model has significantly improved, demonstrating that residual correction effectively enhances prediction accuracy while maintaining model interpretability.

In summary, the BRB-ARR model significantly reduces model complexity through the introduction of an adaptive rule reduction mechanism while maintaining or even improving prediction accuracy. With the integration of the posterior correction mechanism, the model performance is further enhanced, validating the effectiveness and superiority of the proposed method in prediction tasks for complex industrial systems.

**Figure 9:**
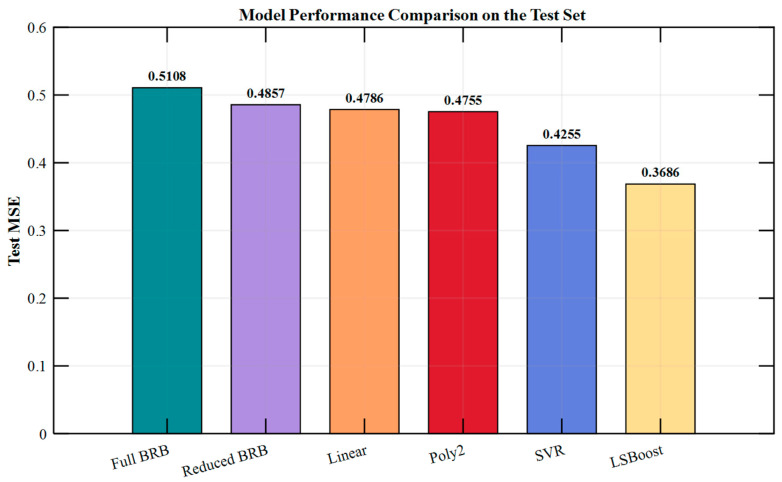
Comparison of model prediction results.

To verify the effectiveness of the posterior correction mechanism in the proposed BRB-ARR model, multiple sets of comparative experiments were conducted on the oil pipeline dataset, and the results are illustrated in Figure 9. In this figure, the x-axis represents different predictive models, including the Full BRB model, Reduced BRB model, and several posterior residual calibration strategies, while the y-axis represents the corresponding test-set MSE values. The numerical labels above each bar denote the specific MSE result of each model.

As shown in Figure 9, the MSE of the BRB model is effectively reduced following rule reduction. Building on this, the introduction of linear and quadratic polynomial error compensation does not yield significant performance improvements, suggesting that simple functional error correction has a limited capacity to characterize complex nonlinear residuals. Furthermore, with the introduction of the SVR-based residual learning method, the test set MSE decreases to 0.4255, demonstrating that data-driven residual modeling can effectively increase the predictive accuracy. However, when LSBoost is employed as the posterior residual correction method, the model performance improves even more significantly, with the test set MSE decreasing to 0.3686, achieving the best performance among all compared methods.

Notably, the posterior correction mechanism proposed in this study performs external modeling and correction of prediction residuals without altering the original BRB rule structure. Consequently, while improving predictive accuracy, this method preserves the semantic structure and inference logic of the original rule base, thereby balancing model performance and interpretability. This characteristic is not inherent to purely data-driven models, such as SVR or LSBoost, when they are used in isolation.

In summary, the BRB-ARR model significantly enhances predictive performance while reducing model complexity through a collaborative mechanism of adaptive rule reduction and posterior residual learning correction. Among these, LSBoost performs best as a posterior correction method. It not only achieves the minimum prediction error but also maintains the transparency of the rule inference structure while enhancing the predictive performance, effectively unifying high precision with the interpretability of the BRB-ARR model.

The source of performance improvement can be attributed to the synergistic effect of three core stages. First, through adaptive rule reduction, the model eliminates redundant knowledge units with minor contributions to the output. This reduces the mean squared error from 0.5291 to 0.4856 in the petroleum pipeline case. This improvement originates from the effective suppression of overfitting risks as the streamlined rule base focuses the inference logic on core features. This process enhances the generalization capability of the model on unseen data. Second, dimensionality reduction compresses the parameter search space from 338 to 122. This significantly lowers the nonlinear complexity of optimization and allows the CMA ES algorithm to converge more stably toward the global optimum. Finally, the posterior calibration module contributes the most significant accuracy gain, further reducing the error from 0.4856 to 0.3619. This demonstrates that LSBoost can capture the systematic residuals caused by rule discretization and compensate for the information loss induced by structural simplification in the output space. Through this mechanism, the model breaks through the accuracy limits of conventional model driven methods.

To further satisfy the ablation requirement for the core modules, a residual calibration experiment combining the BRB model with LSBoost was introduced without changing the original experimental structure, denoted as BRB-LSBoost. The purpose of this experiment is to examine whether the posterior calibration module itself can independently improve the performance of the basic BRB model. The experimental results are reported in Table 7.

The results show that directly applying the LSBoost-based residual calibration module to the original BRB model does not necessarily lead to stable performance improvement. When the residual compensation module is directly applied to the original full-rule BRB, the improvement in test performance is unstable. This is mainly because the full-rule BRB still contains a large number of low-contribution or redundant rules, and strong coupling exists among the high-dimensional parameters. As a result, the model residuals contain not only compensable systematic prediction bias, but also unstable error information caused by rule redundancy, local optimization fluctuations, and sample noise. In this case, the LSBoost residual learner may capture unstable residual patterns and may produce compensations with incorrect directions or excessive magnitudes during the testing stage, thereby weakening the generalization ability of the model.

In contrast, adaptive rule reduction first removes low-contribution rules and reduces the parameter dimensionality, thereby decreasing the interference of redundant rules on the inference results and making the residual structure of the reduced BRB smoother, more stable, and easier to learn. Therefore, the residual compensation module can exert a more stable calibration effect on the basis of the Reduced BRB and achieve more significant accuracy improvement. This indicates that the superior performance of the complete BRB-ARR model is not contributed solely by the posterior calibration module, but results from the synergistic effect of adaptive rule reduction, reduced-dimensional parameter re-optimization, and posterior residual calibration.

The proposed BRB-ARR model is further compared with representative existing BRB-based models on the same oil pipeline leakage case to evaluate its overall performance in terms of prediction accuracy and structural complexity [31]. proposed five potential indicators for quantifying the complexity of BRB systems, including the number of rules (NOR), length of rules (LOR), number of antecedent attributes (NOA), total number of referential values of antecedent attributes (TNRA), and number of consequent referential levels (NOCR). These indicators are inherent structural properties of a BRB system and are usually determined during the knowledge-base design stage. In general, larger values of these indicators imply higher structural complexity of the BRB system. Among them, the setting of referential values not only affects the scale and complexity of the BRB rule base, but also directly influences the model’s ability to characterize the system state space and its modeling effectiveness. Since this study mainly focuses on the balance between structural compactness and prediction accuracy after rule reduction, the NOR and the TNRA are selected as the main basis for measuring model complexity. The model complexity is calculated as follows.

(18) Complexity=NOR×TNRA

This complexity measure has been widely used in previous BRB-based pipeline leakage detection studies, as it can comprehensively reflect the influence of both the rule-base scale and the referential-value structure on model complexity. Based on the same data partition and evaluation metrics, the comparison results of different BRB-based models are summarized in Table 8.

As shown in Table 8, the proposed BRB-ARR model retains only approximately 20 rules on average and achieves an MSE of 0.3619. Compared with the original full-rule BRB containing 56 rules, BRB-ARR significantly compresses the rule base. Since the total number of antecedent referential values is 15, its structural complexity is reduced to 294, which is much lower than those of BRB-r and BRB-IR. Although BRB-r obtains the lowest MSE among the compared models, it retains all 56 rules and has the highest structural complexity. This indicates that its accuracy improvement relies on a larger rule base and a higher parameter optimization burden. In contrast, BRB-ARR achieves better prediction accuracy than GBRB, BRB-DI, and BRB-IR while maintaining relatively low complexity. In particular, compared with BRB-DI, BRB-ARR achieves both lower MSE and lower complexity, demonstrating that the proposed adaptive rule reduction, reduced-dimensional re-optimization, and posterior residual calibration mechanisms can more effectively balance prediction accuracy and model compactness.

Overall, BRB-ARR does not improve prediction performance by increasing the number of rules. Instead, it adaptively removes low-contribution rules, re-optimizes the retained core rule subset, and further calibrates residual bias, thereby achieving a favorable balance between prediction accuracy and rule-base compactness.

To further validate the effectiveness and superiority of the proposed BRB-ARR model, several typical data-driven models are selected for experimental analysis. Under the same dataset and experimental conditions, a back propagation neural network (BP), support vector regression (SVR), and K-nearest neighbors (KNN) are introduced as comparative models. The experimental results are shown in Table 9.

The overall results in Table 9 indicate that the BRB-ARR model exhibits superior performance across multiple evaluation metrics. The MSE of the BRB-ARR model is 0.3619, and the RMSE metric shows a consistent trend, further indicating that the model possesses stronger error control capabilities. In terms of fitting effectiveness, the $R^{2}$ of the BRB-ARR model reaches 0.9386, which is superior to those of the SVM and BPNN and comparable to that of the KNN, suggesting that the model can better characterize data variation trends.

Furthermore, regarding the MAE metric, although the BRB-ARR model outperforms the SVM and BPNN, it is slightly better than the KNN, indicating that there is still room for improvement in local error control. However, from the perspective of overall stability and interpretability, the VAF of the BRB-ARR model reaches 93.91%, the highest among all the models, demonstrating a significant advantage in overall variance explanation capability. In summary, the BRB-ARR model outperforms traditional data-driven models in terms of comprehensive performance, maintaining high prediction accuracy while balancing stability and generalizability, which validates its effectiveness and superiority in safety prediction tasks for complex industrial systems.

### 4.4. Cross-Scenario Validation

In the experimental design of this study, full consideration was given to the adaptability of the model to different operating states, disturbance levels, and input combinations, so that the experimental validation is not limited to accuracy evaluation on a single task, but further reflects the transferability and robustness of the model in industrial safety prediction. To verify the transferability and robustness of the proposed model in industrial safety prediction tasks, this section introduces an additional case study in which the BRB-ARR model is applied to the prediction of structural safety states in liquid-propellant launch vehicles for generalization validation [36].

The second case study does not represent transfer learning across tasks. Instead, it serves as an independent validation under a different industrial operating scenario.

From the perspective of the experimental object, the structural safety problem of liquid-propellant launch vehicles exhibits typical characteristics of industrial safety prediction. Its operating environment is complex, the potential consequences of failure are severe, and significant nonlinear mapping relationships often exist between monitoring variables and safety states. Therefore, this scenario provides a representative experimental basis for validating the generalized applicability of the model in high-risk and complex systems.

To strictly evaluate the generalization ability of the method across different industrial tasks, a structural safety prediction case for liquid propellant launch vehicles was conducted. This task focuses on low frequency structural dynamics under complex loads, which differs from the high frequency vibration signals in bearing data. During the experiment, environmental temperature and humidity remained stable, while inclination angle and vibration frequency had a direct effect on structural response. These two dynamic variables were selected as core input features to increase model sensitivity to risk disturbances. To make the model structure fit the new data distribution, reference values for each attribute were set based on data features and expert knowledge as shown in Table 10. This ensures the rule base covers the full operational range. The model structure was built for the rocket task and parameters were re-optimized using CMA-ES to match the specific sensitivity of the rocket structure. The dataset contains 515 samples, with 445 used for training and 70 for testing. Average results over 10 runs are reported in Table 7. The consistent performance across different tasks proves that the proposed BRB framework is portable and general.

Since the proposed BRB-ARR model involves stochastic optimization, repeated experiments were conducted to reduce the influence of random initialization and stochastic search. Specifically, 10 independent runs were performed under the same data partition and experimental settings. For each run, the model was independently initialized and optimized, and the final performance was evaluated on the testing data. The mean and standard deviation of the evaluation metrics were reported to provide a more reliable statistical assessment of model performance.

Table 11 shows that the proposed BRB-ARR model exhibits superior overall performance to the conventional BRB model in the task of predicting the structural safety state of liquid-propellant launch vehicles [19]. First, the MSE of BRB-ARR is only 0.00029, whereas that of the conventional BRB model is 0.0523, indicating that the proposed model can more accurately characterize the nonlinear mapping relationship between the input features and the structural safety state, thereby substantially reducing prediction bias. Meanwhile, the prediction accuracy of BRB-ARR reaches 98.57%, which is higher than the 95.71% achieved by the conventional BRB model, further demonstrating that the proposed method has stronger discriminative capability and higher predictive reliability in complex industrial safety scenarios.

Second, BRB-ARR also shows a clear advantage in terms of structural compactness. The number of rules is reduced from 441 in the conventional BRB model to 109, and the parameter dimensionality is decreased from 1766 to 438. These results indicate that the adaptive rule reduction mechanism can effectively eliminate redundant rules and low-contribution knowledge units, thereby significantly compressing the model scale. Notably, the substantial reduction in model complexity does not lead to any degradation in performance. On the contrary, improvements are achieved in both prediction error and accuracy, which suggests that the proposed method successfully unifies structural optimization and predictive performance enhancement while preserving the interpretability of BRB inference.

Overall, these results fully demonstrate the effectiveness of the adaptive rule reduction strategy, the secondary optimization in the reduced-dimensional parameter space, and the posterior consistency calibration mechanism introduced in the BRB-ARR model. Rule reduction alleviates knowledge-base redundancy and reduces the burden of parameter optimization; reduced-dimensional optimization reconstructs the collaborative relationships among the core retained rules; and posterior calibration further compensates for the systematic bias that may be introduced by structural simplification. Through their combined effect, the model achieves significant improvements in predictive accuracy, robustness, and deployment efficiency while maintaining transparent inference and interpretability. Therefore, the excellent performance of BRB-ARR in the case of liquid-propellant launch vehicle structural safety prediction not only verifies the effectiveness of the proposed method in this specific scenario, but also further demonstrates its strong generalization potential and application value in complex industrial safety prediction tasks.

For further validation of model performance, comparisons are conducted with SVM, KNN and BPNN under the same experimental setup. The average results of 10 repeated experiments are summarized in Table 12.

As shown in Table 12, for the structural safety state prediction of liquid launch vehicles, the proposed BRB-ARR model achieves an MSE of only 0.00029, which is significantly lower than those of SVM, KNN, and BPNN. This result confirms its superior prediction accuracy and error control ability, ensuring stable inference under limited samples and multi-factor coupling disturbances. Furthermore, the BRB-ARR model attains an average prediction accuracy of 98.57%, with improvements of 1.49%, 1.78%, and 2.50% over SVM, KNN, and BPNN, respectively, demonstrating more reliable decision support for high-risk industrial safety prediction tasks.

In summary, the BRB-ARR model outperforms SVM, KNN, and BPNN in both MSE and accuracy. It not only delivers lower prediction errors and higher recognition precision but also maintains robust inference under constraints of limited samples, partial distribution shift, and multi-factor coupling, fully validating its practical value and technical advantages in industrial safety prediction.

### 4.5. Experimental Summary

Experiments systematically verified the proposed BRB-ARR model on an oil pipeline dataset. The results indicate that the model exhibits excellent comprehensive performance in terms of prediction accuracy, model complexity, and interpretability.

First, by introducing an adaptive rule reduction mechanism based on sensitivity analysis, redundant rules with minor contributions to the model output were effectively eliminated. This reduced the number of rules from 56 to approximately 20 and the optimization dimensionality from 338 to 122, significantly compressing the model structure and lowering computational complexity. During this process, the model’s predictive performance did not decrease but actually improved, verifying the effectiveness of the rule reduction mechanism in enhancing the generalization capability. Building on rule reduction, a posterior residual correction mechanism was introduced to further improve prediction accuracy. Compared with linear and polynomial compensation methods, the LSBoost-based residual learning method can more effectively characterize complex nonlinear errors, further reducing the test set MSE from 0.4862 (after reduction) to 0.3641 and significantly outperforming the other comparative methods. Moreover, compared with typical data-driven models such as SVR, KNN, and BPNN, the proposed model demonstrates superior or more stable performance across multiple evaluation metrics.

Second, the proposed model is validated using a typical case concerning structural safety state prediction for liquid launch vehicles. Experimental results demonstrate that the model outperforms the conventional BRB model and baseline methods including SVM, KNN, and BPNN across all key performance indicators, with a MSE of only 0.00029 and an average prediction accuracy of 98.57%. In summary, the BRB-ARR model achieves both high prediction accuracy and strong robustness, providing an efficient and interpretable solution for safety prediction in high-risk complex industrial systems, and thus exhibits considerable engineering application value.

Furthermore, the proposed method achieves a balance between performance and interpretability at the structural design level. From the perspective of model construction, the BRB-ARR model inherits the inherent rule-driven characteristics of BRB, enabling the inference process to proceed in a sequential manner from rule activation to weight allocation and finally to result aggregation. This process itself possesses strong semantic interpretability. On this basis, the introduced rule reduction mechanism effectively decreases the size of the rule base by eliminating redundant or low-contribution rules, thereby making the model structure more compact. The reduction in the number of rules not only lowers computational complexity but also highlights the importance of each participating rule, resulting in a clearer and more understandable decision-making pathway, which facilitates interpretation of the model behavior from a mechanistic perspective.

On the other hand, the posterior correction mechanism in the BRB-ARR model adopts a structurally decoupled design, treating rule-based inference and posterior correction as relatively independent processes. Without altering the original BRB inference structure or its semantic representation, the model performs validation and compensation of posterior residuals to further improve prediction accuracy. This approach avoids interfering with the original rule semantics, allowing the model to enhance predictive performance while preserving the interpretability of the rule-based reasoning process. From the perspective of interpretability, the core decision-making process is still governed by the rule base, while the correction component functions only as an auxiliary adjustment module. Its role boundary is clearly defined and does not weaken the interpretability at the rule level.

Therefore, the interpretability of the BRB-ARR model can be qualitatively reflected in the following aspects: (1) the inference process is based on explicit rules, exhibiting a clear logical structure; (2) the simplified structure after rule reduction facilitates the identification of key influencing factors; (3) the posterior correction mechanism does not alter the original inference semantics, ensuring that the model maintains a rule-centered interpretability framework.

In summary, the BRB-ARR model significantly improves predictive performance while reducing model complexity, and maintains desirable interpretability at the structural level, demonstrating its effectiveness and application potential in safety prediction tasks of complex industrial systems.

## 5. Conclusions

In this research, a BRB-ARR model that combines adaptive rule reduction and posterior residual correction mechanisms is proposed to address existing issues in industrial safety prediction. Through experimental validation on an oil pipeline dataset, the comprehensive performance of the model regarding prediction accuracy, computational efficiency, and interpretability was systematically evaluated. Compared with traditional data-driven models, the BRB-ARR model exhibits superior or more stable performance across multiple evaluation metrics and possesses stronger interpretability, providing more reliable support for industrial system safety prediction.

Although the proposed BRB-ARR model achieved satisfactory experimental performance on the current datasets, several limitations still exist. First, the experiments in this study were mainly conducted under relatively fixed data distributions, and the adaptability of the model under dynamic operating condition changes, data distribution drift, and unknown fault patterns still requires further validation. Second, as the number of input attributes and referential values further increases, the rule scale may still grow rapidly. Although the proposed adaptive rule reduction mechanism can effectively reduce redundant rules, the model may still face relatively high optimization complexity in larger-scale scenarios. In addition, the posterior residual calibration module relies on the residual distribution learned from historical samples, and its calibration stability may be affected when operating conditions change significantly. Future work will focus on more efficient rule generation and adaptive update mechanisms to improve the adaptability of the model in complex adaptive industrial environments. Additionally, multi-source heterogeneous data fusion and uncertainty modeling methods are introduced to enhance robustness and stability. Finally, the proposed method is validated and expanded across a wider range of engineering application scenarios to further assess its generalizability and practical potential.

## Figures and Tables

**Table 1:** Knowledge Distribution.

Reference Level	NVL	NL	NGL	NM	NS	NVS	PS	PM
Flow Diff	−10	−5	−3	−1	0	1	2	3
**Reference Level**	**NL**	**NM**	**NS**	**VS**	**S**	**PM**	**PL**	
Press Diff	−0.1	−0.05	−0.002	0	0.02	0.05	0.1	

**Table 2:** Average 10-rounds comparison results of BRB and BRB-ARR.

Model Name	MSE	RMSE	R^2^	MAE	VAF
BRB-ARR	0.3619	0.6015	0.9386	0.2231	93.91%
BRB	0.5291	0.7274	0.9103	0.3351	91.38%

**Table 3:** Model comparison of the standard deviation of each indicator.

Model Name	MSE Std	RMSE Std	R^2^ Std	MAE Std	VAF Std
BRB-ARR	0.0107	0.0089	0.0018	0.0074	0.18%
BRB	0.0107	0.0073	0.0018	0.0162	0.11%

**Table 4:** Comparison of computational efficiency between models.

Model Name	Training Time (s)	Inference Time (s)
BRB-ARR	33.4276	0.010284
BRB	46.5412	0.027372

**Table 5:** Statistical Summary of Frequently Retained Rules Across 10 Repeated Experiments.

Rule No.	FlowDiff	PressureDiff	Retention Frequency
5	NVL	S	100%
12	NL	S	100%
19	NGL	S	100%
10	NL	NS	90%
11	NL	VS	90%
33	NS	S	90%
47	PS	S	90%
3	NVL	NS	80%
4	NVL	VS	80%
16	NGL	NM	80%
17	NGL	NS	80%
24	NM	NS	80%
26	NM	S	80%
31	NS	NS	80%
38	NVS	NS	80%
2	NVL	NM	70%
23	NM	NM	70%
39	NVS	VS	70%
40	NVS	S	70%

**Table 6:** Average 10-rounds results with 50% training data proportion.

Model Name	BRB-ARR	Reduced BRB	Full BRB
MSE	0.3619	0.4856	0.5291
Rule	20	20	56
Parameter dimensionality	122	122	338

**Table 7:** Average performance comparison results over 10 rounds for BRB-LSBoost.

Model Name	MSE	RMSE	R^2^	MAE	VAF
BRB-LSBoost	3.3111	1.4512	0.4385	1.1667	64.38%
**Model Name**	**MSE Std**	**RMSE Std**	**R^2^ Std**	**MAE Std**	**VAF Std**
BRB-LSBoost	4.3211	1.1572	0.7327	1.2879	40.58%

**Table 8:** Comparison of Accuracy and Structural Complexity Among Different BRB-Based Models on the Oil Pipeline Leakage Case.

Model	MSE	NOR	TNRA	Complexity
BRB-ARR	0.3619	20	15	300
BRB-r [32]	0.3466	56	15	840
BRB-DI [33]	0.4370	25	15	375
BRB-IR [34]	0.4391	56	15	840
GBRB [35]	0.3988	21	10	210

**Table 9:** Comparative Experimental Results.

Model Name	BRB-ARR	SVM	KNN	BPNN
MSE	0.3619	0.5803	0.4118	0.4287
RMSE	0.6015	0.7575	0.6380	0.6413
R^2^	0.9386	0.9110	0.9374	0.9341
MAE	0.2231	0.3684	0.2259	0.2964
VAF	93.91%	91.12%	93.76%	93.75%

**Table 10:** Reference values of liquid rocket data.

Index	Swing Angle	Vibration Frequency	Index	Swing Angle	Vibration Frequency
1	3.1	0.020	12	9.0	0.055
2	3.5	0.025	13	9.5	0.060
3	4.0	0.028	14	50	0.063
4	5.0	0.030	15	55	0.065
5	5.5	0.035	16	56	0.070
6	6.0	0.038	17	58	0.075
7	6.5	0.040	18	60	0.078
8	7.0	0.045	19	62	0.080
9	7.5	0.048	20	64	0.085
10	8.0	0.050	21	66	0.090
11	8.5	0.053			

**Table 11:** Mean Results of 10 Repeated Experiments for BRB and BRB-ARR.

Model Name	BRB-ARR	BRB
MSE	0.00029	0.0523
Accuracy	98.57%	95.71%
Rule	109	441
Parameter dimensionality	438	1766

**Table 12:** Average Results of 10 Experimental Runs for Different Prediction Models.

Model Name	BRB-ARR	SVM	KNN	BPNN
MSE	0.00029	0.03622	0.03194	0.03325
Accuracy	98.57%	97.08%	96.81%	96.07%

## Data Availability

Data will be made available on request.

## References

[1] Yan X., Xu Y., Jia M. (2021). Intelligent Fault Diagnosis of Rolling-Element Bearings Using a Self-Adaptive Hierarchical Multiscale Fuzzy Entropy. Entropy.

[2] Gholaminejad A., Jorkesh S., Poshtan J. (2023). A comparative case study between shallow and deep neural networks in induction motor’s fault diagnosis. IET Sci. Meas. Technol..

[3] Che Z., Peng C. (2024). Improving Support Vector Regression for Predicting Mechanical Properties in Low-Alloy Steel and Comparative Analysis. Mathematics.

[4] Al-Andoli M.N., Tan S.C., Sim K.S., Seera M., Lim C.P. (2023). A Parallel Ensemble Learning Model for Fault Detection and Diagnosis of Industrial Machinery. IEEE Access.

[5] Puthanveettil Madathil A., Luo X., Liu Q., Walker C., Madarkar R., Qin Y. (2025). A review of explainable artificial intelligence in smart manufacturing. Int. J. Prod. Res..

[6] Kasilingam S., Yang R., Singh S.K., Farahani M.A., Rai R., Wuest T. (2024). Physics-based and data-driven hybrid modeling in manufacturing: A review. Prod. Manuf. Res..

[7] Guo Z., Wang T., Ta Y., Buyao Y., Xie J., Chen J. (2025). Dynamic-Constrained PINN for Complex Machinery System Digital Twin Modeling and Fault Diagnosis.

[8] Feng C., Ye H., Li W., Huang Z., Li M., Chen J., Xu X. (2026). A hybrid model for tool wear monitoring via physics-based and data-driven utilizing unscented Kalman Filter. Adv. Eng. Inform..

[9] Wang Z., Li G., Zhou X., Zhang H., Lin Z., Jia S. (2024). Dynamic analysis of deep groove ball bearing with localized defects and misalignment. J. Sound Vib..

[10] Li L., Wei C., Ma H. (2025). A novel wavelet constrained physics-informed neural network for bearing fault diagnosis. J. Adv. Mech. Des. Syst. Manuf..

[11] Jiao L., Geng X., Pan Q. (2019). Compact Belief Rule Base Learning for Classification with Evidential Clustering. Entropy.

[12] Lian Z., Zhou Z., Zhang X., Feng Z., Han X., Hu C. (2023). Fault Diagnosis for Complex Equipment Based on Belief Rule Base with Adaptive Nonlinear Membership Function. Entropy.

[13] Yin X., He W., Cao Y., Ma N., Zhou G., Li H. (2024). A new health state assessment method based on interpretable belief rule base with bimetric balance. Reliab. Eng. Syst. Saf..

[14] Song H., Yuan Y., Wang Y., Yang J., Luo H., Li S. (2024). A Security Posture Assessment of Industrial Control Systems Based on Evidential Reasoning and Belief Rule Base. Sensors.

[15] Kabir S., Islam R.U., Hossain M.S., Andersson K. (2020). An Integrated Approach of Belief Rule Base and Deep Learning to Predict Air Pollution. Sensors.

[16] Mai J., Huang H., Wei F., Yang C., He W. (2025). Autonomous underwater vehicle fault diagnosis model based on a deep belief rule with attribute reliability. Ocean Eng..

[17] Tang L., Liu L., Li G., Li S., Hu G. (2026). A novel fault diagnosis method for complex systems based on belief rule base and average causal effect. Inf. Sci..

[18] Cheng X., Liu S., He W., Zhang P., Xu B., Xie Y., Song J. (2022). A Model for Flywheel Fault Diagnosis Based on Fuzzy Fault Tree Analysis and Belief Rule Base. Machines.

[19] Sun M., Long X., Xu B., Ding H., Wu X., Yang W., Zhao W., Liu S. (2025). Review of Thrust Regulation and System Control Methods of Variable-Thrust Liquid Rocket Engines in Space Drones. Actuators.

[20] Wang Z., Li S., He W., Yang R., Feng Z., Sun G. (2022). A New Topology-Switching Strategy for Fault Diagnosis of Multi-Agent Systems Based on Belief Rule Base. Entropy.

[21] Cao Y., Zhou Z.J., Hu C.H., Tang S.W., Wang J. (2021). A new approximate belief rule base expert system for complex system modelling. Decis. Support Syst..

[22] Yang L.H., Qian B.Y., Huang C.X., Ye F.F., Hu H., Wu H.D. Predicting Remaining Useful Life of Lithium-Ion Battery Using Extended Belief Rule Base Model. Proceedings of the 2023 18th International Conference on Intelligent Systems and Knowledge Engineering (ISKE).

[23] Wen B., Xiao M., Wang G., Yang Z., Li J., Chen X. (2021). A Fusion Prognostic Method for Remaining Useful Life Prediction Based on an Extended Belief Rule Base and Particle Filters. IEEE Access.

[24] Zhao Y., Wang T., Wang X. (2026). Graph neural networks and belief rule base collaborative modeling for automated and interpretable fault diagnosis in proton exchange membrane fuel cells. PLoS ONE.

[25] Gao F., Bi W. (2023). A fast belief rule base generation and reduction method for classification problems. Int. J. Approx. Reason..

[26] Yang L.-H., Ye F.-F., Liu J., Wang Y.-M. (2023). Belief rule-base expert system with multilayer tree structure for complex problems modeling. Expert Syst. Appl..

[27] Si Z., Qu Y., Shen J., Xing L., Mu M. Rule Reduction for Belief Rule Base Based on Redundancy. Proceedings of the 2024 11th International Conference on Behavioural and Social Computing (BESC).

[28] Zhang Z., Deng Q., He W., Yang C. (2024). A New Method Based on Belief Rule Base with Balanced Accuracy and Interpretability for Student Achievement Prediction. Mathematics.

[29] Martín-Gómez A.M., Ávila-Gutiérrez M.J., Lama-Ruiz J.R., Aguayo-González F. (2024). Industrial Metabolism: A Multilevel Characterization for Designing Sustainable Manufacturing Systems. Machines.

[30] Xu D.-L., Liu J., Yang J.-B., Liu G.-P., Wang J., Jenkinson I., Ren J. (2007). Inference and learning methodology of belief-rule-based expert system for pipeline leak detection. Expert Syst. Appl..

[31] You Y., Sun J., Guo Y., Tan Y., Jiang J. (2022). Interpretability and accuracy trade-off in the modeling of belief rule-based systems. Knowl.-Based Syst..

[32] You Y., Sun J., Jiang J., Lu S. (2020). A new modeling and inference approach for the belief rule base with attribute reliability. Appl. Intell..

[33] Han P., Zhang Q., He W., Chen Y., Zhao B., Li Y., Zhou G. (2024). A double inference engine belief rule base for oil pipeline leakage. Expert Syst. Appl..

[34] Sun C., Yang R., He W., Zhu H. (2022). A novel belief rule base expert system with interval-valued references. Sci. Rep..

[35] Chen H., He W., Zhou G., Cui Y., Gao M., Qian J., Liang M. (2024). A novel game-based belief rule base. Expert Syst. Appl..

[36] He W., Cheng X., Zhao X., Zhou G., Zhu H., Zhao E., Qian G. (2023). An interval construction belief rule base with interpretability for complex systems. Expert Syst. Appl..

